# Two centipedes new to the fauna of La Palma (Canary Islands, Spain) and notes on the Lithobiomorpha of the island (Chilopoda, Myriapoda)

**DOI:** 10.3897/BDJ.6.e26746

**Published:** 2018-07-11

**Authors:** Nesrine Akkari, Anne-Sarah Ganske

**Affiliations:** 1 Natural History Museum Vienna, Vienna, Austria; 2 University of Vienna, Department of Integrative Zoology, Vienna, Austria

**Keywords:** The Canary Islands, centipedes, *
Lithobius
*, taxonomy, new records

## Abstract

Recent collecting on the island of La Palma has yielded two new records, Lithobius (Monotarsobius) crassipesoides and Lithobius (Lithobius) melanops, the former also being new to the Canary Islands and is recorded for the first time after its recent description. We additionally provide species records for the lithobiomorph species of the island, with new locality data and a distribution map based on recent and literature records. We update the checklist of the lithobiomorph species of the Canaries and document all the newly collected species.

## Introduction

The Lithobiomorpha of the Canary Islands is known to encompass 3 genera and 14 species. Among these, three belong to the genus *Lamyctes* Meinert, 1868 and the rest is placed in the genus *Lithobius* Leach, 1814 (Bonato et al. 2016; Eason 1985, Eason 1996; Eason and Enghoff 1992; Serra 1984). Five of these species are endemic to the Canary Islands, *viz.* *Lithobius (Lithobius) teneriffae* Latzel, 1895; *Lithobius (Monotarsobius) canariensis* Eason, 1992; *Lithobius (Monotarsobius) consimilis* Eason, 1992; *Lithobius (Monotarsobius) gomerae* Eason, 1985 and *Lithobius (Monotarsobius) speleovolcanus* Serra, 1984. Whereas the highest species diversity is recorded in Gran Canaria and Tenerife with eight and nine species respectively, the fauna of La Palma is only known from four species, namely Lithobius (L.) pilicornis Newport, 1844; Lithobius (L.) lusitanus Verhoeff, 1925; Lithobius (L.) obscurus Meinert, 1872 and Lithobius (M.) crassipes L. Koch, 1862. A list of the Canarian species and their respective distributions across the islands was provided by Eason and Enghoff (1992).

La Palma is among the most volcanically active and one of the smallest of the Canary Islands. Located at north-western most part of the Archipelago, it is dominated by the Caldera de Taburiente in the North and the Cumbre Vieja in the South, with the ridge Cumbre Nueva connecting both parts. During a recent field trip on the island, we were able to collect a number of centipedes, mainly of the order Lithobiomorpha, among which two new records for the island, Lithobius (L.) melanops Newport, 1845 and Lithobius (M.) crassipesoides Voigtländer, Iorio, Decker & Spelda, 2017, the latter also being recorded for the first time in the Canary Islands.

Additional localities are provided for the hitherto known species and a distribution map, based on older and recent records, is presented to illustrate the occurrences of the different species on the island.

## Material and methods

All specimens were collected by hand or obtained by sifting in October 2017 and kept in 96% ethanol in the collections of the Naturhistorisches Museum Wien. Multifocus images were obtained with a Nikon SMZ25 stereomicroscope equipped with a Nikon DS-F2.5 camera using NIS-Elements Microscope Imaging Software with an Extended Depth of Focus (EDF) patch. Some images were edited in Photoshop CS6 and figures assembled in Adobe InDesign CS6. The map was produced in QGIS 2.18.19, using the coordinate reference system WGS84 and topographic data obtained from USGS (2006) Shuttle Radar Topography Mission, 3 Arc Second scene SRTM_f03_p208r040, Filled Finished-A 2.0, Global Landcover Facility, University of Maryland, College Park, Maryland, February 2000.

## Results

**Order Lithobiomorpha**

**Family Lithobiidae**

**Genus *Lithobius* Leach, 1814**

### Lithobius (Monotarsobius) crassipes L. Koch, 1862

**Material**: No new records.

**Distribution**: Widespread in Europe. Also recorded in North Africa, Turkey. See Bonato et al. (2016) for a complete list of occurrences.

**Remarks**: The record of *L. crassipes* from La Palma was first provided by Eason and Enghoff (1992), based on adult specimens from Roque de Los Muchachos at 2300 m. The same authors also mention a number of differences observed between the specimens from the Canaries with their European counterparts.

### Lithobius (Lithobius) lusitanus Verhoeff, 1925

Fig. 1a

**Material**: 1 male (NHMW9320), road to Roque de los Muchachos, 28°43'22.30"N, 17°46'55.40"W, 958 m, mixed forest, wet habitat, under stones and logs, leaf litter, 28.10.2017, leg. Akkari N. & Ganske A.-S.; 1 male (NHMW9323), road to Roque de los Muchachos, path to Pico de la Nieve, 28°43'58.20"N, 17°49'19.30"W, 1898 m, pine forest, under stones and leaf litter, 28.10.2017, leg. Akkari N. & Ganske A.-S.

**Distribution**: Known to be endemic to the island La Palma. Hitherto recorded from Cubo de la Galga, Los Tilos and Cumbre Nueva (Eason 1985; Eason and Enghoff 1992).

### Lithobius (Lithobius) obscurus Meinert, 1872

**Material**: No new records.

**Distribution**: Açôres; Bermuda; Ecuador; Madagascar; Morocco; Spain including the Canary Islands; Peru; Uruguay (Bonato et al. 2016).

**Remarks**: Other records from La Palma include specimens from Santa Cruz and Cumbrecita (Eason 1985, Eason and Ashmole 1992).

### Lithobius (Lithobius) pilicornis Newport, 1844

Fig. 1b

**Material**: 2 males (NHMW9324, NHMW9325), 2 females (NHMW9326, NHMW9327), Pico de la Nieve, road to Roque de los Muchachos, 28°43'21.50"N, 17°47'11.40"W, 1027 m, walking path, pine forest, 01.11.2017, leg. Akkari N.; 1 male (NHMW9317), road to Roque de los Muchachos, 28°43'22.30"N, 17°46'55.40"W, 958 m, mixed forest, wet habitat, under stones and logs, leaf litter, 28.10.2017, leg. Akkari N. & Ganske A.-S.; 1 female (NHMW9321), Caldera de Taburiente, 28°45'46.70"N, 17°46'29.70"W, 402 m, mixed forest, 30.10.2017, leg. Akkari N.

**Distribution**: Açôres; Canary Islands; Madeira; Nicobar Islands; Corsica; France; Great Britain; Ireland; Italy; Netherlands; Portugal; Sardegna; Spain; Switzerland (Bonato et al. 2016). In the Canaries, the species is known from Lanzarote; Gran Canaria; Tenerife and La Palma (Eason and Enghoff 1992).

**Remarks**: Eason and Enghoff (1992) gave the first record of the species from La Palma, based on an unspecified number of specimens from Cumbre Nueva.

### Lithobius (Monotarsobius) crassipesoides Voigtländer, Iorio, Decker & Spelda, 2017 new record

Figs 2, 3

**Material**: 1 female (NHMW9315), road to Roque de los Muchachos, path to Pico de la Nieve, 28°43'58.20"N, 17°49'19.30"W, 1898 m, pine forest, under stones and leaf litter, 28.10.2017, leg. Akkari N. & Ganske A.-S.

**Descriptive notes**: The female specimen we found fits the species description of Lithobius (Monotarsobius) crassipesoides from Spain as provided by Voigtländer et al. (2017). Colour: light brownish to yellowish. Head: 1.07 mm long, 1.16 mm wide; antennae: 20 antennal articles, 2.12 mm long; Ocelli in two rows with a slightly larger posterior ocellus: 8 (1+4, 3) on the left and 9 (1+5, 3) on the right; coxosternum with a narrow and deep median diastema. Body length: 9.35 mm. Leg-pairs 9 and 10 and the left leg 15 are missing. Plectrotaxy as in Table 1. The specimen from La Palma is missing Vmt and VaP on the 13^th^ legs and has only VmF on the 15^th^ leg in comparison to the specimens from the mainland (see Voigtländer et al. 2017, p. 28)

**Distribution**: Spain (Navarre, Gipuzkoa); The Canary Islands (La Palma).

**Remarks**: This represents the first record of the species from La Palma and the Canary Islands.

### Lithobius (Lithobius) melanops Newport, 1845 new record

Figs 4, 5

**Material**: 1 male (NHMW9322), Los Tilos, 28°47'5.70"N, 17°48'18.40"W, 601 m, walking path, mixed forest, sifting, under stones, in litter, 31.10.2017, leg. Akkari N. & Solodovnikov A.; 2 males (NHMW9318, NHMW9319), La Laguna, 28°48'34.94"N, 17°48'34.94"W, 865 m, artificial water reservoir, along an agricultural path, under stones, 01.11.2017, leg. Akkari N.; 2 females (NHMW9316), La Laguna, 28°48'34.94"N, 17°48'34.94"W, 865 m, artificial water reservoir, along an agricultural path, under stones, 01.11.2017, leg. Akkari N.; 1 female (NHMW9314), Los Tilos, 28°47'25.30"N, 17°48'16.40"W, 433 m, gorge, waterfall, very wet soil, under stones, in litter, 31.10.2017, leg. Akkari N. & Solodovnikov A.

**Distribution**: Known to occur only in Tenerife, *L. melanops* is here recorded for the first time on La Palma. The species occurrence on the island is most probably due to anthropogenic activities.

### Updated List of the Lithobiomorpha from the Canary Islands (those from La Palma are marked with an asterisk)

*Lamyctes (Lamyctes) emarginatus* (Newport, 1844)

*Lamyctes*(*Lamyctes*) *coeculus* (Brölemann, 1889)

*Lithobius (Lithobius) lusitanus* Verhoeff, 1925 *

*Lithobius (Monotarsobius) gomerae* Eason, 1985

*Lithobius (Lithobius) teneriffae* Latzel, 1895

*Lithobius (Monotarsobius) crassipes* L. Koch, 1862 *

*Lithobius (Lithobius) lapidicola* Meinert, 1872

*Lithobius (Monotarsobius) consimilis* Eason, 1992

*Lithobius (Monotarsobius) speleovolcanus* Serra, 1984

*Lithobius (Lithobius) melanops* Newport, 1845 *

*Lithobius (Monotarsobius) canariensis* Eason, 1992

Lithobius (Monotarsobius) crassipesoides Voigtländer, Iorio, Decker & Spelda, 2017 *

*Lithobius (Lithobius) obscurus* Meinert, 1872 *

*Lithobius (Lithobius) pilicornis* Newport, 1844 *

Lamyctes (Metalamyctes) albipes (Pocock, 1895)

## Discussion

With the two new records of *L. melanops* and *L. crassipesoides*, the lithobiomorph fauna of La Palma presently includes 6 species, all belonging to the genus *Lithobius* and, among which, two are from the subgenus Monotarsobius. The finding of *L. crassipesoides* is particularly interesting as this represents the first record of the species from the Canaries and as a whole after its recent description (Voigtländer et al. 2017). These same authors provided a detailed molecular and morphological comparison of the species with its close congener and widespread species *L. crassipes*, based on specimens from Spain, France and Germany, respectively. Perhaps among the most striking morphological differences are a DaP spine starting from the leg-pair 12, a narrower middle coxosternal notch and somewhat more slender female gonopods (Voigtländer et al. 2017, fig. 16). These characters have been verified on the female specimen newly found in La Palma (Fig. 3). This begs the question whether the previous findings of *L. crassipes* from La Palma and in the Canary Islands is rather referring to *L. crassipesoides*.

The second new record is represented by *L. melanops*, a widespread species known from Africa, Europe and northern America (see Bonato et al. 2016 for detailed distribution records). *L. melanops* was hitherto recorded exclusively from Tenerife in the Canary Islands (Latzel 1895; Eason 1985). Eason (1985) further mentioned that the specimens he then studied agree with the description provided by Serra (1984). Latzel (1895) mentioned in his original description of the species from Tenerife (as *Lithobius orotavae* Latzel, 1895) an additional paramedian pair of prosternal teeth but these were not present on the specimens from La Palma (Fig. 4b).

**Figure 6.**
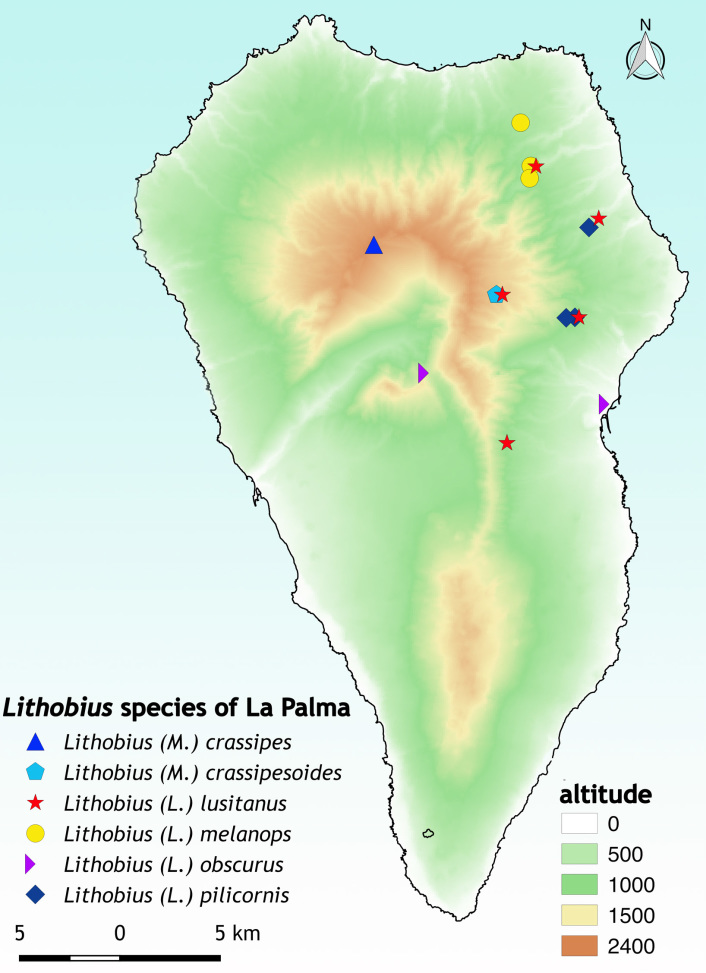
Distribution map illustrating the occurrences of *Lithobius* species on La Palma based on new and literature records (Eason 1985; Eason and Enghoff 1992).

**Figure 7.**
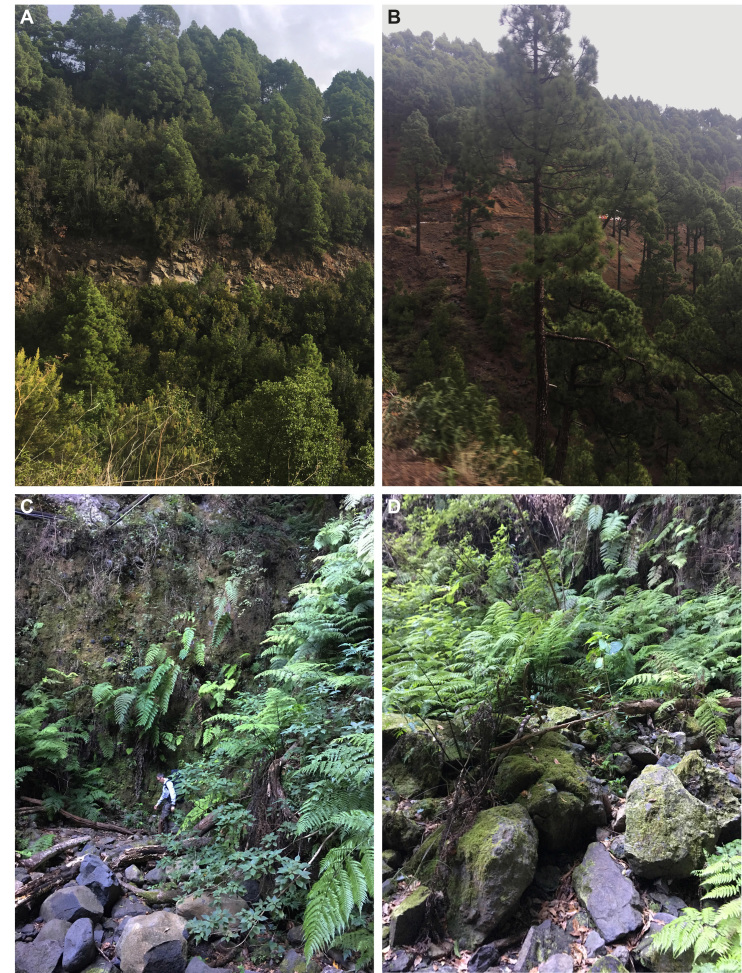
Example of habitats accommodating *Lithobius* species; **A, B.** Pine forest along the road to Roque de los Muchachos; **C, D.** Laurel Forest, near a waterfall Los Tilos.

The species of the genus *Lithobius* are mainly distributed in the north-eastern part of La Palma (Fig. 6). This sector has probably been more explored for invertebrates in general due to the interesting and varied types of habitats ranging from dry pine forests to wet mixed forests (see Fig. 7) and perhaps is more suitable for myriapods in comparison with the dry south-western part.

## Figures and Tables

**Figure 1a.**
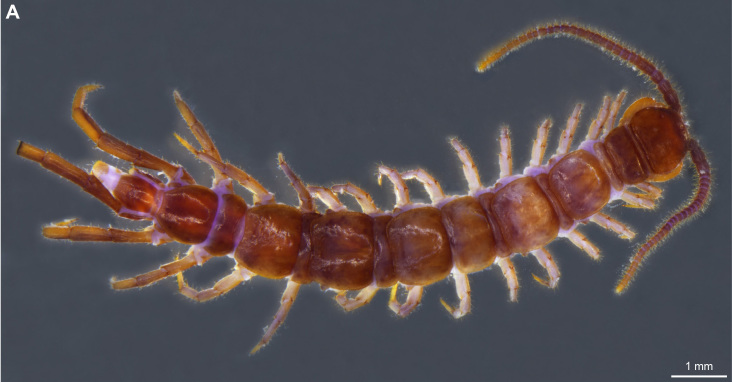
Lithobius (Lithobius) lusitanus, habitus, dorsal view, adult male NHMW9320.

**Figure 1b.**
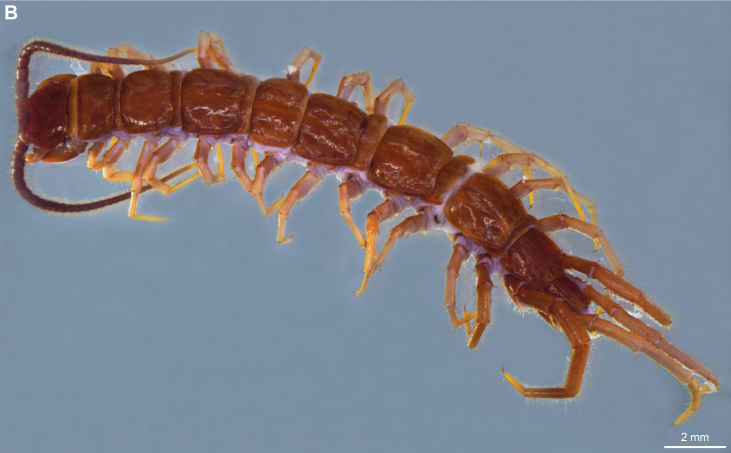
Lithobius (Lithobius) pilicornis, habitus, dorsal view, adult female NHMW9327.

**Figure 2a.**
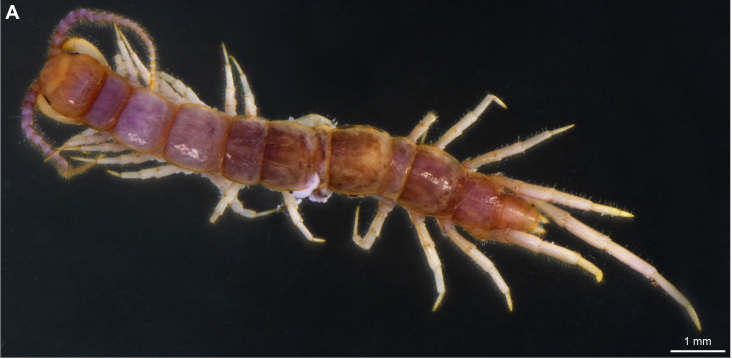
Habitus, dorsal view.

**Figure 2b.**
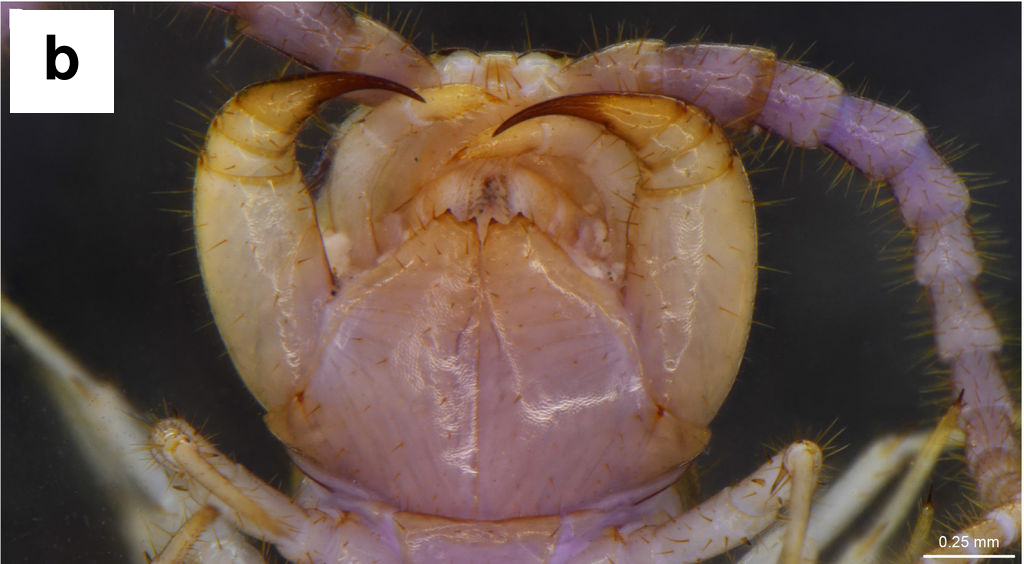
Head, ventral view.

**Figure 3a.**
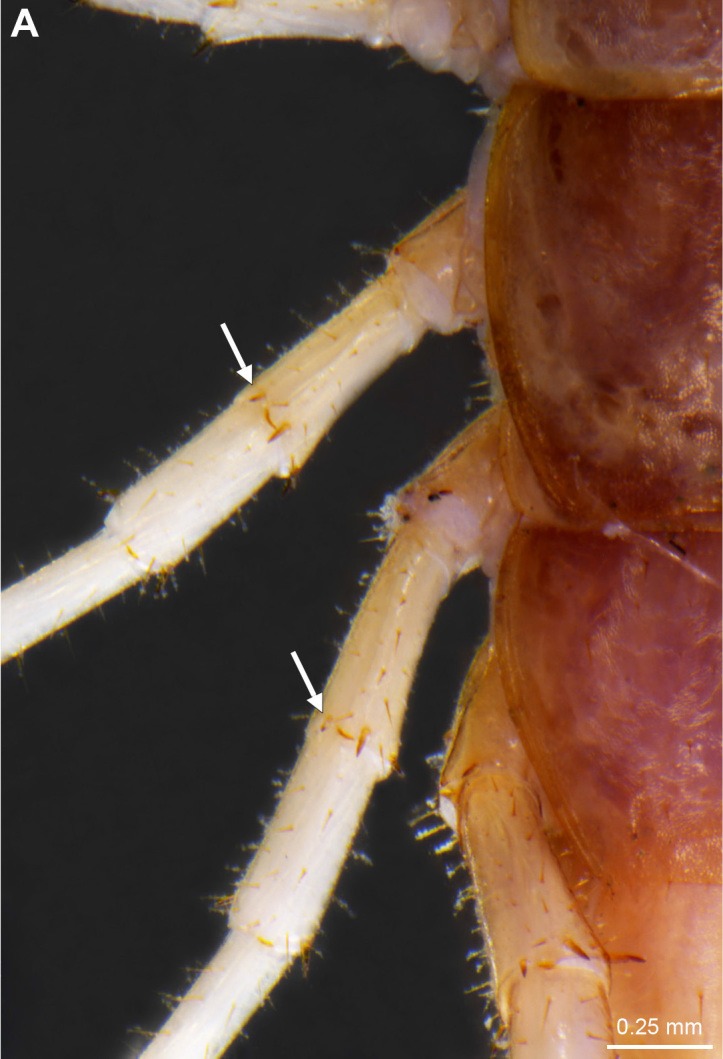
Dorsal anterior spines on 12^th^ and 13^th^ prefemora, arrows.

**Figure 3b.**
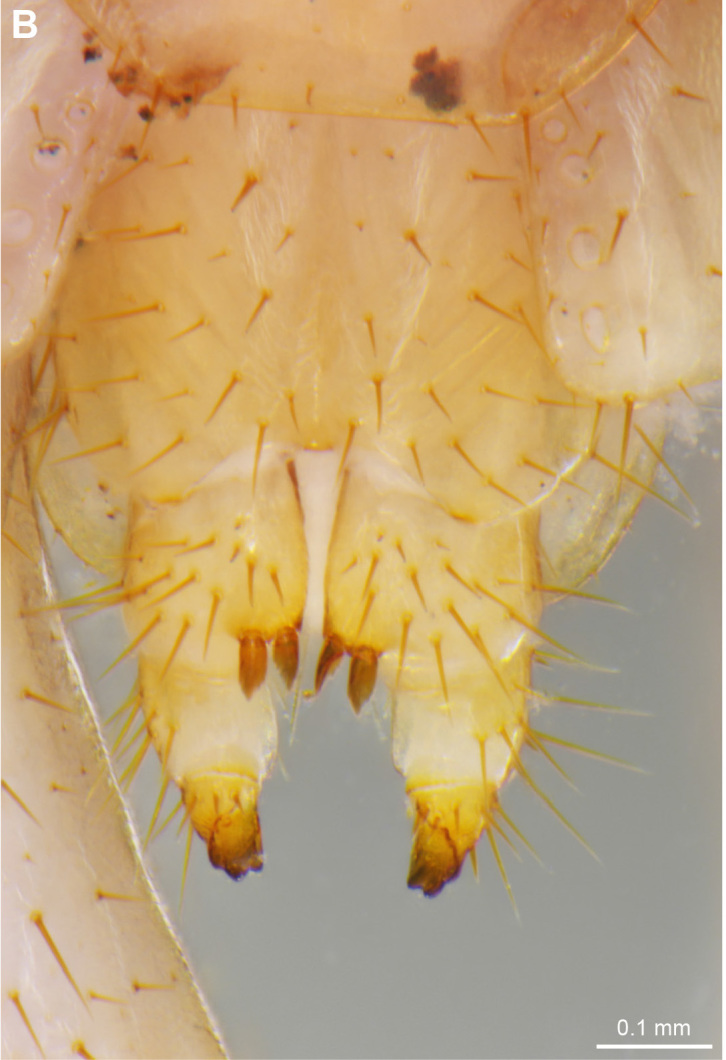
Gonopods, ventral view.

**Figure 4a.**
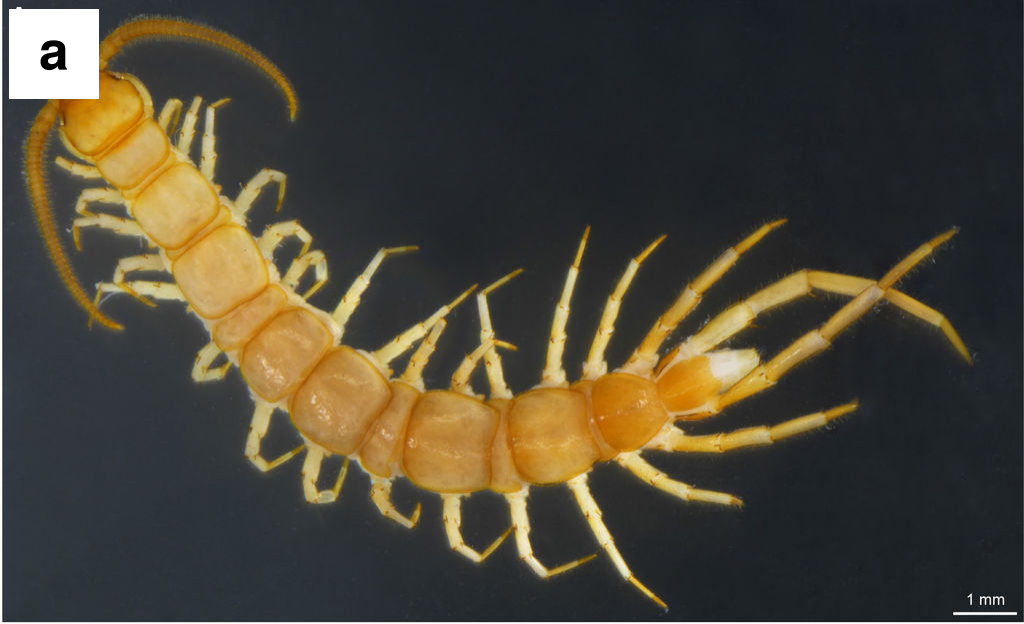
Habitus, dorsal view.

**Figure 4b.**
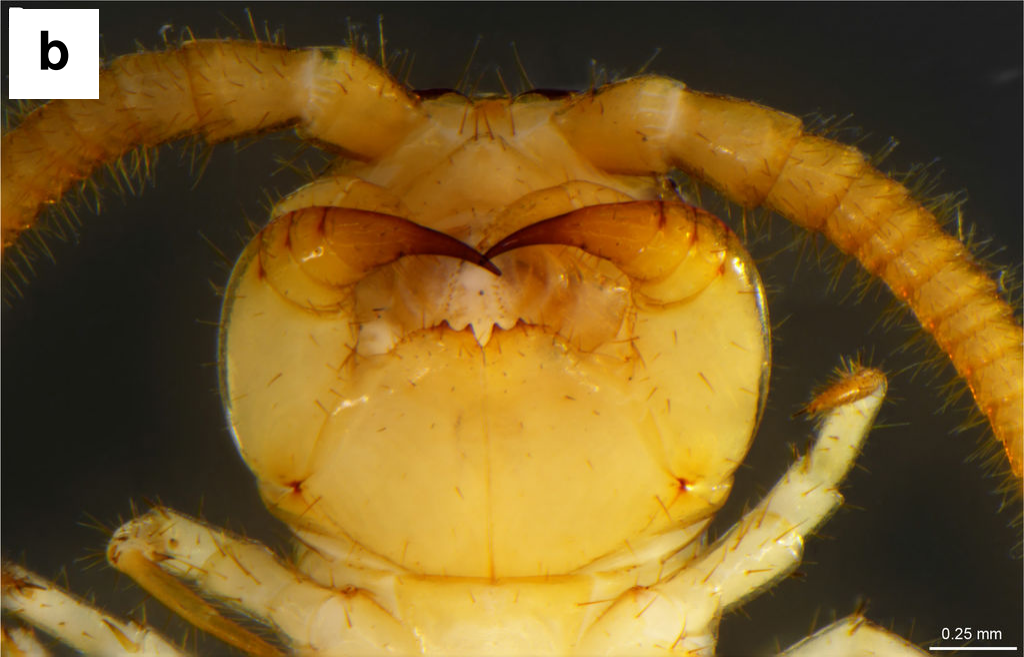
Head, ventral view.

**Figure 5a.**
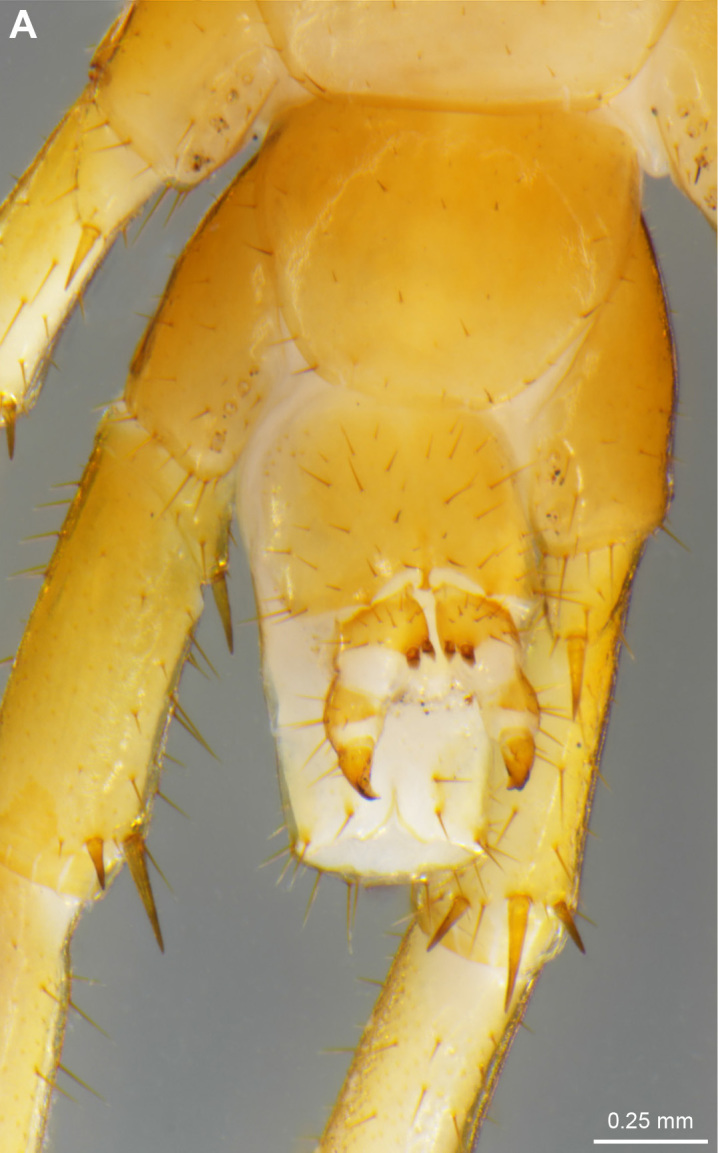
Adult female NHMW9314.

**Figure 5b.**
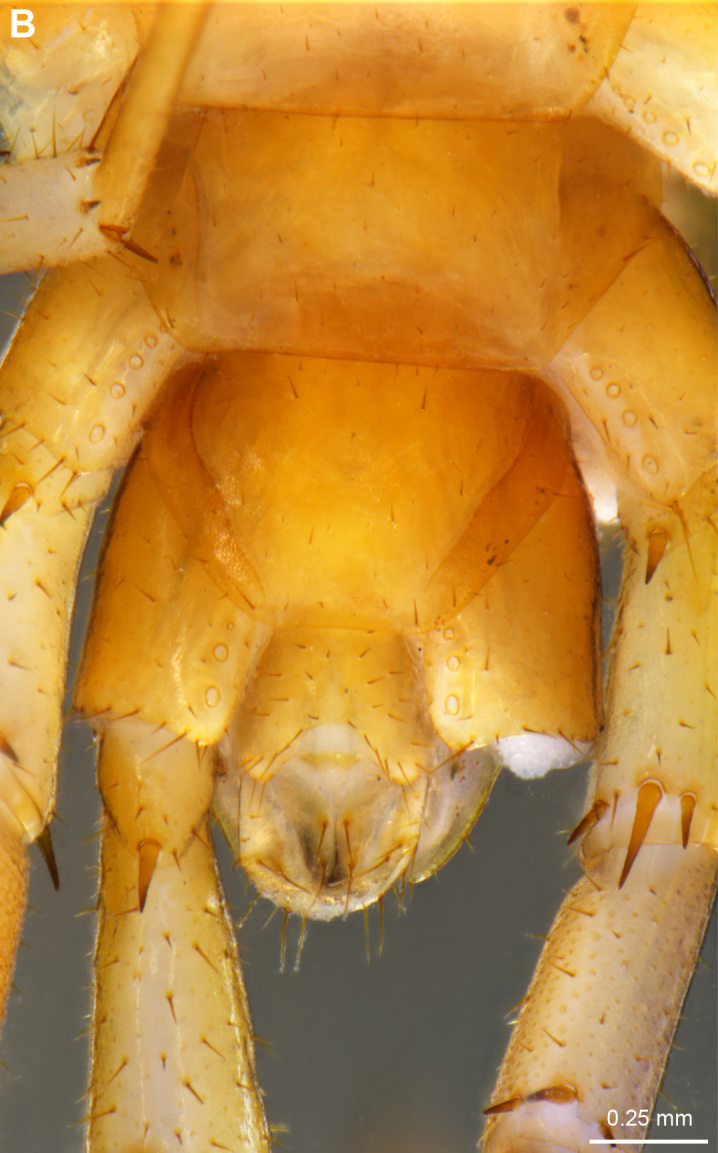
Adult male NHMW9319.

**Table 1.** Table 1. Plectrotaxy of Lithobius (Monotarsobius) crassipesoides, female specimen from La Palma Island (NHMW 9315)

Legpair	Ventral					Dorsal				
	C	t	P	F	T	C	t	P	F	T
1	-	-	p	amp	m	-	-	mp	a	a
2	-	-	p	amp	m	-	-	mp	ap	a
3	-	-	p	amp	am	-	-	mp	ap	ap
4	-	-	p	amp	am			mp	ap	ap
5	-	-	-	amp	am			mp	ap	ap
6	-	-	-	amp	am			mp	ap	ap
7	-	-	p	amp	am			mp	ap	ap
8	-	-	p	amp	am			mp	ap	ap
9	?	?	?	?	?	?	?	?	?	?
10	?	?	?	?	?	?	?	?	?	?
11	-	-	mp	amp	am	-	-	mp	p	ap
12	-	-	mp	amp	am	-	-	amp	p	p
13	-	-	mp	amp	am	a	-	amp	P	p
14	-	m	amp	amp	m	a	-	amp	p	-
15	-	m	amp	m	-	a	-	amp	-	-

## Hosting institution

Natural History Museum Vienna
